# Towards equitable and immersive outdoor orienteering: An artificial intelligence-driven multi-objective route planning framework with augmented sand cat swarm optimization

**DOI:** 10.1371/journal.pone.0344770

**Published:** 2026-03-11

**Authors:** Qingzhu Lun, Boya Li, Yuehui Zhou

**Affiliations:** School of Sports Science, Qufu Normal University, Qufu, Shandong, China; Huazhong University of Science and Technology, CHINA

## Abstract

Outdoor orienteering has emerged as a globally popular recreational activity and competitive sport, combining navigational challenges with physical endurance across diverse natural terrains. Despite its growing popularity, the design of optimal orienteering routes presents significant challenges for recreation planners, requiring careful consideration of both competitive fairness and participant engagement. To address these challenges, this study establishes five fundamental design principles that systematically balance competitive equity with user experience enhancement. Building upon these principles, we develop a novel computational framework that integrates mathematical modeling techniques with intelligent optimization algorithms. Specifically, our methodology reformulates the route design challenge as a constrained multi-objective optimization problem and introduces an enhanced sand cat swarm optimization (SCSO) algorithm for efficient solution generation. Through comprehensive simulations across 50 distinct terrain profiles representing varying levels of complexity, we demonstrate the efficacy of our approach. Quantitative results show consistent performance improvements in route optimality metrics compared to conventional methods, which contribute to both the theoretical understanding of recreational route optimization and practical applications in outdoor activity planning.

## Introduction

Outdoor orienteering originated in Sweden, initially as a military activity before evolving into an outdoor recreation [1]. Participants in outdoor orienteering use a map and a compass to sequentially reach various checkpoints marked on the map, with the fastest participant declared as the winner upon reaching the terminus [2]. Outdoor orienteering challenges the comprehensive application of personal intellect and physical fitness, making it an outdoor recreation that seamlessly blends navigational skills with physical endurance training [3]. In unfamiliar outdoor environment, participants must rely on their sense of direction, map-reading abilities, compass usage, as well as their own critical thinking and judgment to success [4].

Outdoor orienteering integrates the strengths of both outdoor and adventure activities, rendering it a highly appealing type of outdoor recreation. Firstly, outdoor orienteering possesses a kind of advantages akin to outdoor activities, such as promoting health and development [5–7], fostering an awareness of and capability to manage risks [8,9], etc. Additionally, outdoor orienteering shares the benefits associated with adventure activities, such as enhancing intrinsic motivation and self-determination [10], and improving the well-being of participants [11,12], etc.

Orienteering route design (ORD) is essential for outdoor orienteering planning. ORD involves planning the starting point, checkpoints, and terminal point on suitable maps or topographic charts, aiming to create fair routes for outdoor orienteering [13]. Outdoor orienteering typically features several routes with shared starting and terminal points, but distinct checkpoints along different routes, each accommodating one or more participants [14]. For outdoor orienteering planner, ORD presents a considerable challenge. Manual design of outdoor orienteering routes is time-consuming and poses difficulties in ensuring both the justice of the routes and enjoyable experience for participants [15].

To accurately plan outdoor orienteering routes, we can transform ORD into a mathematical problem and solve it using mathematical methods. Nevertheless, ORD is essentially a high-dimensional combinatorial optimization problem with strong non-convexity and multiple coupling constraints. The decision variables include the spatial positions and visiting sequence of checkpoints, while constraints arise from terrain accessibility, route fairness, safety requirements, and competition rules. Moreover, ORD inherently involves trade-offs among multiple objectives, such as route fairness, physical challenge, safety, and overall experience, which further increases the complexity of the solution space. These characteristics lead to a highly irregular and discontinuous search landscape with numerous local optima. Using canonical mathematical methods to solve ORD problem not only demands a significant amount of computational time but also requires substantial computational resources. Deterministic optimization algorithms or gradient-based methods are therefore prone to premature convergence and may easily become trapped in local optima when addressing ORD. Therefore, using intelligent methods is a better way to solve ORD problem. Intelligent methods represent a category of approaches that seek optimal solutions to problems by simulating the operational mechanisms observed in various natural phenomena. These methods are commonly employed to address complex issues [16]. Compared to deterministic algorithms, intelligent methods possess inherent advantages such as stochastic operators for both global and local searches, as well as flexibility, independence, and simplicity in their principles [17]. Numerous commonly employed intelligent methods and their enhancements have emerged, finding widespread application in practical production and daily life [18]. For example, Zhang et al. [19] utilized multi-objective particle swarm optimization (MOPSO) to solve robot path planning problem with uncertain danger sources. Nazarahari et al. [20] presented a hybrid intelligent method for path planning of multiple mobile robots in continuous environments. Patle et al. [21] presented the rigorous study of mobile robot navigation techniques used so far, including genetic algorithm (GA), ant colony optimization (ACO), neural network (NN), particle swarm optimization (PSO) etc. Nonetheless, intelligent methods are rarely applied in ORD. This paper aims to use intelligent methods to realize automatic ORD on the known map and overcome the disadvantages of manual route design.

Nevertheless, traditional intelligent methods are prone to falling into local optima in ORD. Due to the high-dimensional non-convex search space and the strong coupling among constraints and objectives, conventional swarm-based algorithms such as GA, PSO, and ACO may suffer from loss of population diversity and insufficient global exploration capability in later iterations. Therefore, we adopt sand cat swarm optimization (SCSO) algorithm to solve the issue. SCSO is a now nature‑inspired algorithm proposed by Seyyedabbasi et al. [22]. As a recently proposed swarm intelligence optimization algorithm, SCSO exhibits superior optimization performance compared to traditional intelligent algorithms [23]. It possesses strong optimization capabilities and fast convergence speeds, among other notable features [24–26]. Therefore, this paper employs SCSO algorithm to solve the mathematical problem of ORD.

The remainder of this paper consists of three major parts: Methodology, Results and Conclusion. In the part of Methodology, we propose five principles of outdoor ORD, and transform the five principles into mathematical formulations. Then we describe SCSO algorithm and present the procedure of ORD using SCSO algorithm. At last of this part, we introduce the simulation experiments of this paper. In the part of Results, we compare the performance among different intelligent methods using statistical results, and present route designed by different methods which showing the good performance and robustness of SCSO in outdoor ORD. In the part of Conclusion, we summarized the research results of this paper, and summed up the contributions of this paper for outdoor ORD.

## Methodology

### Principles of outdoor orienteering route design (ORD)

Ensuring both the justice of the routes and enjoyable experience for participants is the core principle of ORD. For the convenience of outdoor orienteering planner, we formulated five principles for ORD based on the core philosophy, aiming to provide better practical guidance. The first three principles aim to ensure justice in ORD, while the latter two focus on enhancing experience of participants in orienteering.

a) Principle 1: Uniform route length distribution

Variability in route lengths may create unequal challenges, potentially compromising competition fairness. Uniform length distribution ensures participants face comparable physical demands while facilitating objective performance evaluation by judges and spectators.

b) Principle 2: Consistent checkpoint spacing

Non-uniform distributions of checkpoints can result in varying difficulty levels for participants as they navigate through the route. Excessive distance between adjacent checkpoints may lead to mental fatigue among participants, hindering their ability to perform optimally in outdoor orienteering. Therefore, ensuring uniform distances between adjacent checkpoints is important in the justice of outdoor orienteering.

c) Principle 3: Equivalent elevation gain

Terrain roughness, particularly elevation changes, significantly impacts participant speed and endurance. Uniform elevation gain across routes ensures equitable physical demands while minimizing disproportionate wear and tear on participants.

d) Principle 4: Dispersed checkpoint placement

Checkpoints are specific locations that participants must pass through during the competition. Geographic dispersion of checkpoints prevents route redundancy, where participants might otherwise retrace similar paths. Strategic distribution enhances navigational diversity, thereby improving the competitive experience.

e) Principle 5: Minimum route separation

Maintaining adequate distance between parallel routes serves dual purposes: preventing physical congestion during events and eliminating opportunities for unauthorized inter-participant communication, thereby preserving both competitive fairness and participant experience.

### Mathematical formulations of route design principles

In order to accurately design outdoor orienteering routes according to the five design principles, we model these principles as five objective functions, transforming the ORD problem into a multi-objective optimization problem, which can then be addressed using intelligent methods.

Let *N* present the number of routes, and $R_{i}=\left\{r_{i1},r_{i2},...,r_{in_{i}}\right\}$ present the checkpoints sequence of the *i*-th route, where *r*_*ij*_ presents the checkpoint number of the *j*-th checkpoint in the *i*-th route, and *n*_*i*_ presents the number of checkpoints in the *i*-th route. Let $n=\underset{0<i<N}{max}\left(n_{i}\right)$ present the maximum number of checkpoints across all routes.

We assume the number of checkpoints along all routes are equal, meaning all *n*_*i*_ (0 < *i* < *n*) are equal and $n_{i}=n=\underset{0<i<N}{max}\left(n_{i}\right)$. In addition, we assume the starting point and terminal point located at the same position, meaning the outdoor orienteering routes are circular routes. Let the starting/terminal point be presented as *r*_0_.

a) Mathematical formulation of Principle 1

Assuming the length between the *j*-th checkpoint and the next checkpoint in the *i*-th route is denoted as *l*_*ij*_*.* Hence the formula for the total length of the *i*-th route is denoted as *l*_*i*_ and given as follows:


$$l_{i}=\sum_{j=0}^{n}l_{ij},0<i<N$$ (1)

where *l*_*i0*_ means the length between the starting/terminal point and the checkpoint *r*_*i1*_, and *l*_*in*_ means the length between the checkpoint *r*_*in*_ and the starting/terminal point.

Let *l*_dr_ presents the demanded route length, therefore the mathematical formulation of *Principle 1* is given as follows:


$$min\;f_{\text{1}}=\frac{\text{1}}{N}\sum_{i=1}^{N}{\left(l_{i}^{error}\right)}^{\text{2}}$$ (2)

$$\begin{array}{c} l_{i}^{error}=\left\{ \;\;\begin{array}{cc} \left|l_{i}-l_{dr}\right|, & \varepsilon_{\text{1}}<\left|l_{i}-l_{dr}\right|\\ 0, & \left|l_{i}-l_{dr}\right|<\varepsilon_{\text{1}} \end{array}\right. \end{array}$$ (3)

where $\varepsilon_{\text{1}}$ is a small relatively small value that denotes the allowance for a minor margin of route length error.

b) Mathematical formulation of Principle 2

Let *l*_dc_ presents the demanded length of adjacent checkpoints, therefore the mathematical formulation of *Principle 2* is given as follows:


$$min\;f_{\text{2}}=\frac{\text{1}}{N}\sum_{i=1}^{N}\sum_{j=0}^{n}{\left(l_{ij}^{error}\right)}^{\text{2}}$$ (4)

$$\begin{array}{c} l_{ij}^{error}=\left\{ \;\;\begin{array}{cc} \left|l_{ij}-l_{dc}\right|\text{,} & \varepsilon_{\text{2}}<\left|l_{ij}-l_{dc}\right|\\ 0\text{,} & \left|l_{ij}-l_{dc}\right|<\varepsilon_{\text{2}} \end{array}\right. \end{array}$$ (5)

where $\varepsilon_{\text{2}}$ is a small relatively small value that denotes the allowance for a minor margin of length error of adjacent checkpoints.

c) Mathematical formulation of Principle 3

Assuming *e*_*i*_ presents the elevation gains of the *i*-th route. Hence the mathematical formulation of *Principle 3* is given as follows:


$$min\;f_{\text{3}}=\frac{\text{1}}{N}\sum_{i=1}^{N}{\left(e_{i}-\overset{―}{e}\right)}^{\text{2}}$$ (6)

where $\overset{―}{e}$ presents the mean value of elevation gains across all routes.

d) Mathematical formulation of Principle 4

Assuming *θ*_*ij*_ presents the angle formed by checkpoint *r*_*ij*_ and its preceding and succeeding checkpoints, with *r*_*ij*_ as the vertex. To ensure more dispersed distribution of checkpoints along the same route, we set *θ*_d_ (*θ*_d_ < 90°) as the demanded minimum angle. Hence the mathematical formulation of *Principle 4* is given as follows:


$$min\;f_{\text{4}}=\frac{\text{1}}{N}\sum_{i=1}^{N}\sum_{j=0}^{n}{\left(\theta_{ij}^{error}\right)}^{\text{2}}$$ (7)

$$\begin{array}{c} \theta_{ij}^{error}\left\{ \;\;\begin{array}{cc} \theta_{d}-\theta_{ij}, & \theta_{ij}<\theta_{d}\\ 0, & \theta_{d}<\theta_{ij}<180^{\circ }-\theta_{d}\\ \theta_{ij}-\left(180^{\circ }-\theta_{d}\right), & 180^{\circ }-\theta_{d}<\theta_{ij} \end{array}\right. \end{array}$$ (8)

where *θ*_*i*0_ means the angle formed by the starting/terminal point in the *i*-th route and its preceding and succeeding checkpoints, with the starting/terminal point as the vertex.

e) Mathematical formulation of Principle 5

Assuming $\left(\overset{―}{x_{i}},\overset{―}{y_{i}}\right)$ presents the average coordinates of all checkpoints including the starting/terminal point along the *i*-th route. Let *p*_d_ presents the demanded minimum direct distance between two routes. Hence the mathematical formulation of *Principle 5* is given as follows:


$$min\;f_{\text{5}}=\frac{\text{2}}{N\left(N-1\right)}\sum_{i=2}^{N}\sum_{k<i}^{N}{\left(p_{ij}^{error}\right)}^{\text{2}}$$ (9)

$$\begin{array}{c} p_{ij}^{error}=\left\{ \begin{array}{cc} p_{d}-p_{ij}, & p_{ij}<p_{d}\\ 0, & p_{d}<p_{ij} \end{array}\right. \end{array}$$ (10)

where $p_{ij}=\sqrt{{\left(\overset{―}{x_{i}}-\overset{―}{x_{j}}\right)}^{\text{2}}+{\left(\overset{―}{y_{i}}-\overset{―}{y_{j}}\right)}^{\text{2}}}$ presents the direct distance between the average coordinates of *i*-th and *j*-th route.

### Mathematical formulation of multi-objective optimization problem

We formulate the ORD problem as a multi-objective optimization task according to the mathematical formulations of the five design principles, and integrate multiple objective functions into a single objective function, given as follows:


$$min\;F=\alpha_{\text{1}}f_{\text{1}}+\alpha_{\text{2}}f_{\text{2}}+\alpha_{\text{3}}f_{\text{3}}+\alpha_{\text{4}}f_{\text{4}}+\alpha_{\text{5}}f_{\text{5}}$$ (11)

where *α*_*i*_ (*i* = 1,…,5) denote non-negative weighting coefficients assigned to each objective function.

The coefficients *α*_*i*_ do not represent physical quantities, but preference parameters that reflect the relative importance of different design principles in route planning. To ensure comparability among objectives with different scales, all objective functions are first normalized to a unified range. The weighting coefficients are then selected within the interval [0,1], subject to. In this study, the weights are determined based on practical planning considerations and further validated through sensitivity analysis to ensure robustness of the optimization results.

### SCSO for outdoor orienteering route design

#### Description of SCSO.

**Fig 1:**
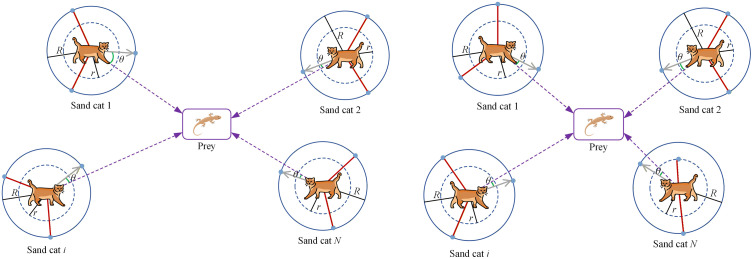
Position updating mechanism of SCSO: (a) Iteration i; (b) Iteration i + 1.

The sand cat swarm optimization (SCSO) algorithm is a metaheuristic algorithm inspired by the natural behavior of sand cat and this algorithm draws inspiration from the remarkable ability of sand cat to detect low-frequency noise [21]. The two primary actions of sand cats are searching (exploration) and attacking (exploitation) prey. The updating mechanism of SCSO is presented in Fig 1. Given the strong resemblance between the predation strategy of sand cats and the optimization process, these actions can be expressed mathematically as follows:

Each sand cat is modelled as a 1 × *D* array $X_{i}=\left\{x_{i1},x_{i2},...,x_{iD}\right\}$ presenting the solution to *D* dimensional optimization problem and the values of the 1 × *D* array $\left\{x_{i1},x_{i2},...,x_{iD}\right\}$ have lower and upper boundaries. If there are *P* sant cats in the population, then the sant cats can be modelled as a *P × D* matrix ${\left[X_{\text{1}}^{T},X_{\text{2}}^{T},...,X_{P}^{T}\right]}^{T}$. The value $\vec{r_{G}}$ presents the general sensitivity range given as follows:


$$\vec{r_{G}}=S_{M}-\left(\frac{S_{M}\times {\text{iter}}_{\text{c}}}{{\text{iter}}_{\text{Max}}}\right)$$ (12)

where *S*_*M*_ is a constant typically set to 2, iter_c_ is the current iteration and iter_max_ is the maximum iterations. The value $\vec{R}$ is an adaptive factor of searching and attacking given as follows:


$$\vec{R}=2\times \vec{r_{G}}\times \text{rand}\left(0,1\right)-\vec{r_{G}}$$ (13)

The value $\vec{r}$ presents the specific sensitivity range of each sand cat given as follows:


$$\vec{r}=\vec{r_{G}}\times \text{rand}\left(0,1\right)$$ (14)

In the searching phase, let $\vec{Pos_{\text{bc}}}$ present the best-candidate position and $\vec{Pos_{\text{c}}}$ present the current position, hence the position update equation is given follow:


$$\vec{Pos}(t+1)=\vec{r\cdot }\left({\vec{Pos}}_{\text{bc}}(t)-\text{rand}(0,1)\cdot {\vec{Pos}}_{\text{c}}(t)\right)$$ (15)

In the attacking phase, let $\vec{Pos_{b}}$ present the best position meaning the best solution and $\vec{Pos_{c}}$ present the current position, hence the position update equation is given follow:


$$\vec{Pos}(t+1)=\vec{Pos_{b}}\left(t\right)-\vec{r}\cdot \vec{Pos_{rnd}}\cdot cos\left(\theta \right)$$ (16)

$$\vec{Pos_{\text{rnd}}}=\left|\text{rand}\left(0,1\right)\cdot \vec{Pos_{\text{b}}}\left(t\right)-\vec{Pos_{\text{c}}}\left(t\right)\right|$$ (17)

Equation (18) illustrates the position update for each sand cat during both the searching phase (exploration) and attacking phase (exploitation).


$$\begin{array}{c} \vec{X}(t+1)=\left\{ \begin{array}{cc} \vec{Pos_{\text{b}}}\left(t\right)-\vec{Pos_{\text{rnd}}}\left(t\right)\cdot cos\left(\theta \right)\cdot \vec{r}, & \text{if}\left|R\right|\leq 1(\text{exploitation)}\\ \vec{r}\cdot \left(\vec{Pos_{\text{bc}}}\left(t)-\text{rand}(0,1)\cdot \vec{Pos_{\text{c}}}(t\right)\right), & \text{if}\left|R\right|>1(\text{exploration)} \end{array}\right. \end{array}$$ (18)

Compared to other intelligent methods, SCSO offers the following advantages:

(1) SCSO algorithm is more successful in solving complex optimization problems compared to other intelligent methods;
(2) SCSO algorithm can escape local optimal traps and exhibits suitable and balanced behavior between exploration and exploitation phases compared to other optimization algorithms;
(3) SCSO algorithm requires fewer parameters and operators compared to other metaheuristic algorithms;

SCSO algorithm is easier to implement compared to other metaheuristic algorithms. Therefore, we select SCSO as the optimization algorithm for the ORD problem.

#### Procedure of ORD using SCSO algorithm.

The procedure of ORD using SCSO algorithm is given follows:

**Step 1** Obtain terrain information of outdoor orienteering map and initialize the population of SCSO algorithm.

**Step 2** Utilize the Equation (11) to calculate the objective function.

**Step 3** If convergence criteria are met or the maximum number of iterations has been reached, terminate the process and select the position with the minimum objective function value as the optimal solution.

**Step 4** Utilize the Equation (18) to update positions of each sand cat.

**Step 5** Update the iteration number iter_c_ ← iter_c_ + 1 and other parameters, then go to **Step 2**.

## Simulation experiments

### Generation of random maps

**Fig 2:**
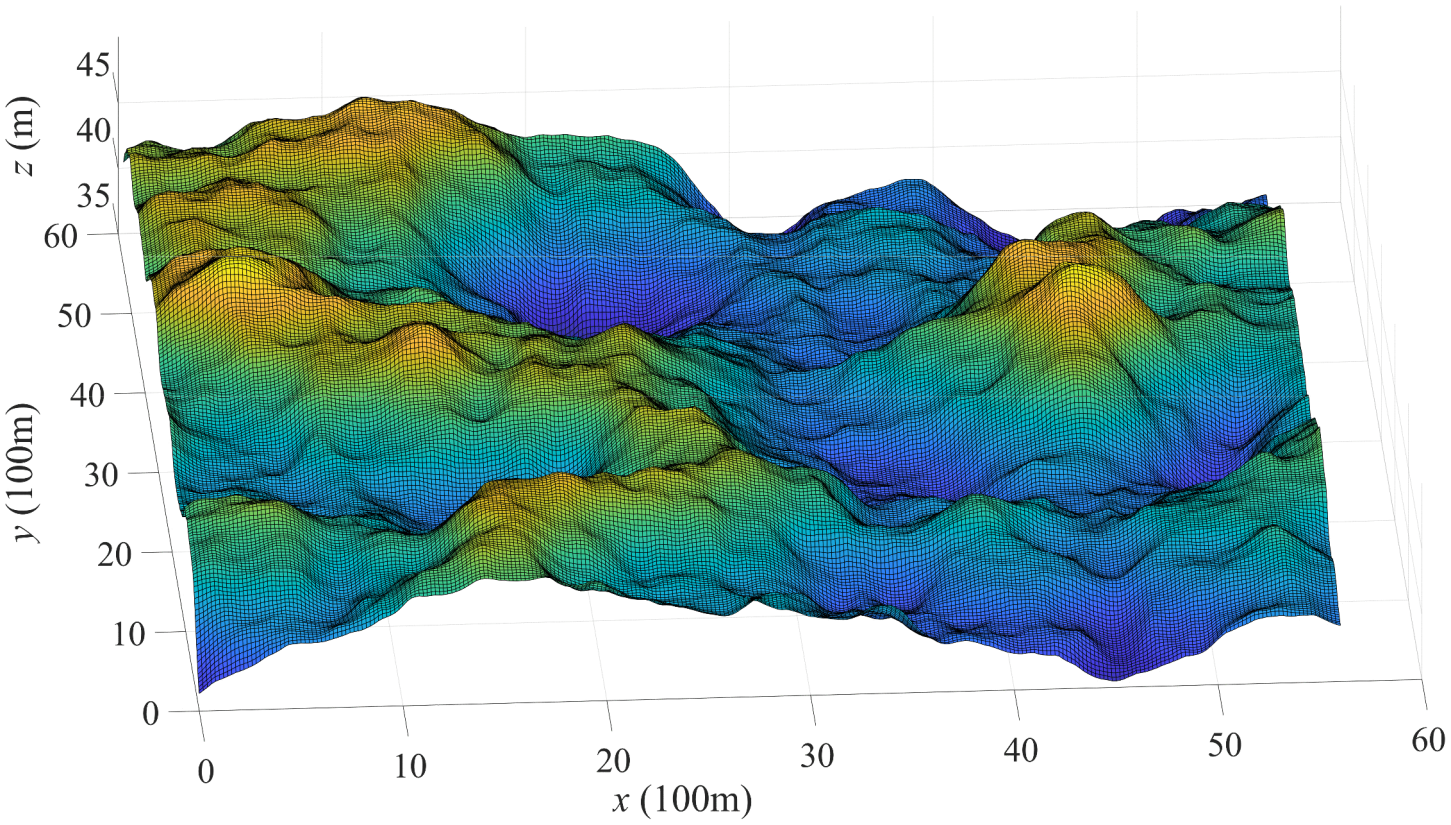
Simulated outdoor orienteering map.

**Fig 3:**
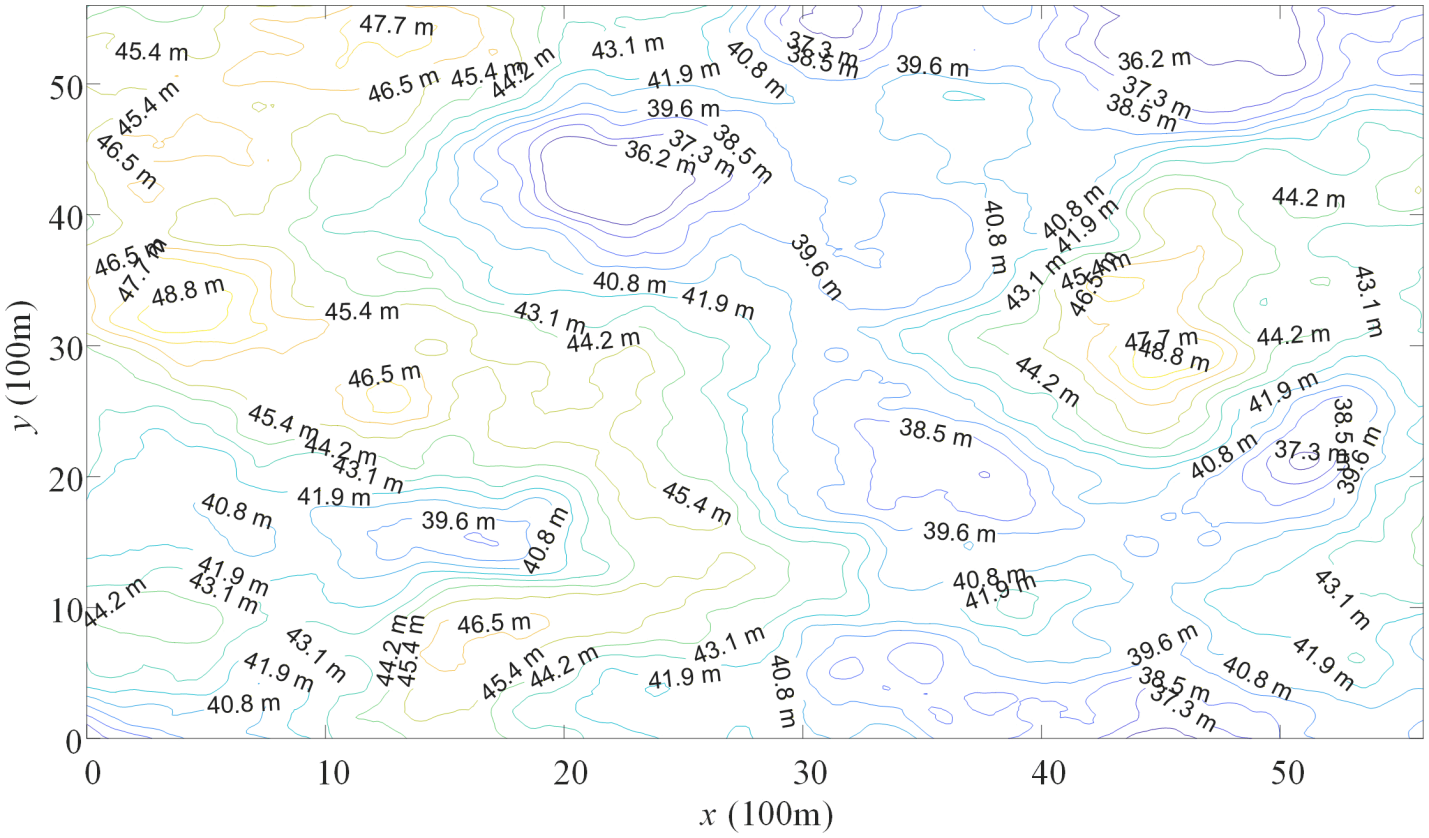
Contour lines of the simulated outdoor orienteering map.

To ensure the rigor and reliability of experiments, we randomly generated 50 different simulated outdoor orienteering maps. One of the simulated outdoor orienteering maps is illustrated in Fig 2, and the contour lines of the map are depicted in Fig 3. For each map, the area is 6000m × 6000m, with employing the superimposition of 2048 randomly placed convex areas to simulate the undulations of the terrain, as shown in Fig 2. Fig 3 presents the terrain in detail height, which can help participants in route planning, navigation, and decision-making in outdoor orienteering.

### Calculation of route length

**Fig 4:**
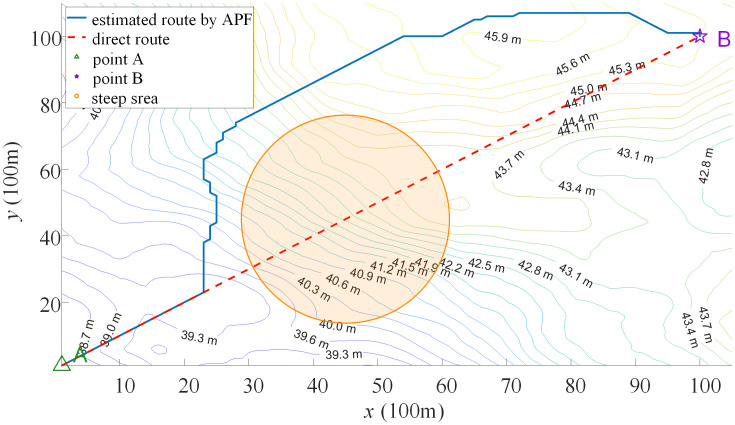
Bias of direct distance in practical scenario.

It is worth noting that in manual ORD, the direct distance between adjacent checkpoints is commonly used as the length of the route between two points. However, this approach can be biased in practical scenarios. For instance, in Fig 4, when there is a steep area (as indicated by the orange area) between two checkpoints as illustrated in point A to point B, participants typically navigate around rather than direct across (as indicated by the dashed red line). If the direct distance between two checkpoints is still used as the length between them, it may result in unfair route design.

To address this issue, we employed the Artificial Potential Field (APF) method to estimate the route of orienteer based on specific terrain. APF is a classical approach to path planning and robot navigation. This method simulates a virtual potential field around the agent to achieve path planning. In this virtual field, the target point generates an attractive force field, while obstacles produce repulsive force fields. The agent is treated as an object subjected to forces, moving within this potential field to find the optimal path. By continuously adjusting the speed and direction, agent navigates through the potential field to eventually reach the target point. APF method is a mature and efficient real-time route planning method which is suitable for many applications in autonomous navigation and path planning (Zhiyang & Tao, 2017). Therefore, we considered the length of the route estimated by APF (as indicated by the solid blue line in Fig 4) as the distance between two checkpoints.

### Setup of simulation experiments

We assume that the starting point and the terminal point are both located at the centre of the map. To demonstrate the superiority of the method employed in this paper, classic PSO algorithm and the newly proposed Dung Beetle Optimizer (DBO) algorithm (Xue & Shen, 2023) are compared with the SCSO algorithm across 50 different terrains. For fair comparison, the population size, maximum iterations and other parameters of SCSO, DBO and PSO are kept identical. Some of the parameters mentioned earlier are as follows: *N* = 5, *n* = 6, *l*_dr_ = 10.5 km, *ε*_1_ = 0.5 km, *l*_dc_ = 1.5 km, *ε*_2_ = 0.3 km, *θ*_d_ = 40°, *p*_d_ = 2.5 km, *α* = 0.9, *β* = 0.8, *γ* = 0.6, *η* = 0.9, *λ* = 0.7, *P* = 50, iter_M_ = 30. The simulation is *p*rogrammed in Matlab 2024b, and the hardware environment for the simulation is a computer with an Intel Core i9-12900K CPU at 3.2 GHz and 32 GB RAM.

## Results

### Statistical results of different intelligent methods

For optimization problems, the value of objective function represents the quality of optimization result. Since this paper is based on an error-designed objective function, lower objective function value indicates better optimization result, meaning the designed orienteering routes are fairer and the participant experience is better. The variation of objective function value with respect to the number of iterations is referred to as objective function curve, which can reflect the optimization speed and result of the intelligent method for the optimization problem. A good objective function curve should have a low final value and a small number of iterations to reach the final value. PSO and DBO are selected as representative classical and recently proposed swarm-based algorithms, providing baseline and advanced comparison references for evaluating the performance of SCSO.

**Fig 5:**
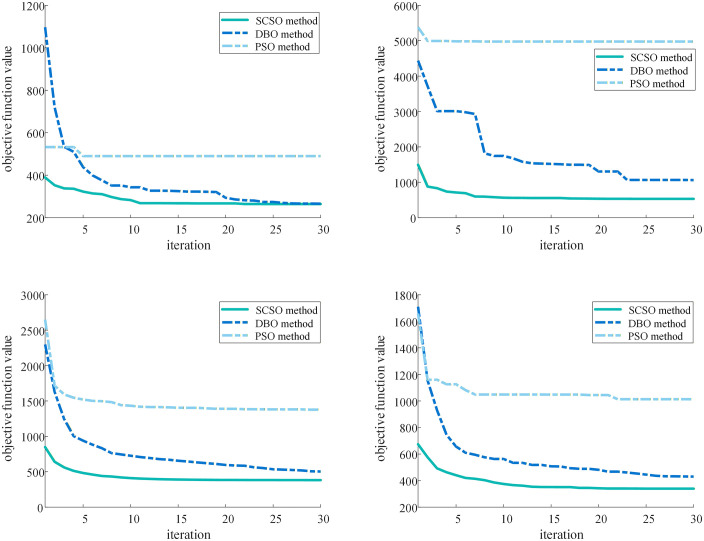
Objective function curves of different intelligent methods: (a) the best objective function curves; (b) the worst objective function curves; (c) the mean of objective function curves; (d) the median of objective function curves.

Fig 5 depicts the statistical results of objective function curves in different simulated maps. The dashed light blue lines present the objective function curves of PSO algorithm, the dashed bule lines present the objective function curves of DBO algorithm, and the dashed green lines present the objective function curves of SCSO algorithm. Fig 5(a) presents the best objective function curves of the three methods in the 50 different simulated maps. This figure shows that SCSO algorithm and DBO algorithm have the lowest final value, whereas SCSO algorithm has smaller number of iterations. These curves mean the design results from SCSO algorithm and DBO algorithm are similar in specific map, yet the required computational resources and computational time of SCSO algorithm are lower.

Fig 5b, 5c and 5d present the worst, mean and median of objective function curves in the 50 different simulated maps, respectively. These figures show that SCSO algorithm has lower final value and smaller number of iterations than other algorithms, meaning SCSO algorithm has better performance and robustness for ORD in different maps.

The detail results of the simulated experiments are shown in Table 1. Justice error is an index to represent the error on justice of the designed routes, which is the weighted sum of the Equations (2), (4) and (6). Smaller value of Justice error represents fairer routes. In 41 out of 50 simulated outdoor orienteering maps, the Justice error values of SCSO algorithm are smaller than other methods, meaning the route designed by SCSO algorithm are fairer than other methods. Experience error is an index to represent the error on participant experience of the designed routes, which is the weighted sum of the Equation (7) and (9). Smaller value of experience error represents better participant experience. In 40 out of 50 simulated outdoor orienteering maps, the experience error values of SCSO algorithm are smaller than other methods, meaning the route designed by SCSO algorithm have better participant experience than other methods. In addition, the weighted sum of Justice error and experience error is the objective function value calculated by Equation (11). In 39 out of 50 simulated outdoor orienteering maps, the objective function values of SCSO algorithm are smallest, meaning SCSO algorithm is better at balancing justice and participant experience in ORD than other methods.

**Table 1:** Detail results of the simulated experiments.

Map	PSO algorithm		DBO algorithm		SCSO algorithm		Best justice	Best Exp.	Best overall
	Justice error	Exp.^a^ error	Justice error	Exp. error	Justice error	Exp. error			
**1**	1253.7	707.3	189.9	104.2	176.1	121.6	**SCSO**	DBO	DBO
**2**	709.0	488.6	185.8	101.0	267.6	202.6	DBO	DBO	DBO
**3**	655.4	455.5	230.8	152.6	202.9	136.7	**SCSO**	**SCSO**	**SCSO**
**4**	771.2	607.1	306.7	246.0	225.9	139.0	**SCSO**	**SCSO**	**SCSO**
**5**	2780.7	2195.4	250.3	181.9	309.8	178.7	DBO	**SCSO**	DBO
**6**	686.6	393.7	201.3	142.1	244.1	136.3	DBO	**SCSO**	DBO
**7**	972.2	781.3	161.7	117.0	270.0	171.6	DBO	DBO	DBO
**8**	603.4	339.6	269.9	163.1	212.3	138.1	**SCSO**	**SCSO**	**SCSO**
**9**	965.8	682.5	264.4	197.3	184.1	140.6	**SCSO**	**SCSO**	**SCSO**
**10**	516.0	350.2	215.8	146.0	212.7	157.8	**SCSO**	DBO	DBO
**11**	875.7	640.9	178.2	130.7	161.8	127.3	**SCSO**	**SCSO**	**SCSO**
**12**	922.0	596.8	272.1	155.1	184.8	108.0	**SCSO**	**SCSO**	**SCSO**
**13**	635.6	377.7	314.8	254.4	248.2	174.3	**SCSO**	**SCSO**	**SCSO**
**14**	419.8	289.1	384.7	272.6	226.6	125.5	**SCSO**	**SCSO**	**SCSO**
**15**	593.0	431.5	601.5	386.2	244.7	146.1	**SCSO**	**SCSO**	**SCSO**
**16**	423.5	269.7	288.4	204.8	247.2	137.0	**SCSO**	**SCSO**	**SCSO**
**17**	660.0	387.0	505.9	276.3	223.5	143.5	**SCSO**	**SCSO**	**SCSO**
**18**	911.0	533.1	262.4	145.5	188.5	110.2	**SCSO**	**SCSO**	**SCSO**
**19**	556.3	447.4	273.9	156.4	272.5	152.6	**SCSO**	**SCSO**	**SCSO**
**20**	869.2	706.2	432.2	318.9	254.2	187.9	**SCSO**	**SCSO**	**SCSO**
**21**	732.7	554.0	319.9	181.4	163.7	100.6	**SCSO**	**SCSO**	**SCSO**
**22**	470.4	316.8	249.5	204.1	195.0	114.4	**SCSO**	**SCSO**	**SCSO**
**23**	492.1	320.5	348.4	244.4	226.7	182.6	**SCSO**	**SCSO**	**SCSO**
**24**	1235.8	894.4	315.9	181.4	232.6	161.5	**SCSO**	**SCSO**	**SCSO**
**25**	985.3	755.2	325.2	263.9	155.6	124.2	**SCSO**	**SCSO**	**SCSO**
**26**	765.2	575.5	306.0	175.9	296.0	236.0	**SCSO**	DBO	DBO
**27**	320.7	211.0	410.5	228.7	295.8	165.3	**SCSO**	**SCSO**	**SCSO**
**28**	595.5	363.7	240.5	134.0	281.8	153.8	DBO	DBO	DBO
**29**	550.8	383.2	275.4	178.4	237.9	143.9	**SCSO**	**SCSO**	**SCSO**
**30**	634.4	465.4	250.8	192.9	219.8	119.2	**SCSO**	**SCSO**	**SCSO**
**31**	687.1	406.1	339.2	258.4	231.7	180.9	**SCSO**	**SCSO**	**SCSO**
**32**	1201.0	865.1	203.1	130.5	271.2	221.2	DBO	DBO	DBO
**33**	1081.7	622.9	280.4	190.3	283.7	185.1	DBO	**SCSO**	**SCSO**
**34**	315.8	239.1	164.3	101.6	221.1	167.5	DBO	DBO	DBO
**35**	815.6	516.9	299.2	174.6	276.9	182.3	**SCSO**	DBO	**SCSO**
**36**	651.5	396.6	318.4	225.7	197.3	138.3	**SCSO**	**SCSO**	**SCSO**
**37**	801.4	535.9	283.7	188.2	322.5	188.0	DBO	**SCSO**	DBO
**38**	1467.2	1110.8	225.8	183.0	196.2	148.3	**SCSO**	**SCSO**	**SCSO**
**39**	746.1	413.3	336.2	273.1	194.2	127.9	**SCSO**	**SCSO**	**SCSO**
**40**	697.9	396.1	216.1	127.2	194.7	133.1	**SCSO**	DBO	**SCSO**
**41**	1108.8	673.5	307.1	167.0	219.9	154.1	**SCSO**	**SCSO**	**SCSO**
**42**	1013.9	724.6	449.4	335.2	213.5	139.5	**SCSO**	**SCSO**	**SCSO**
**43**	616.2	364.4	257.5	210.1	233.0	154.1	**SCSO**	**SCSO**	**SCSO**
**44**	471.2	318.4	320.8	195.4	227.3	154.1	**SCSO**	**SCSO**	**SCSO**
**45**	1754.1	1180.6	336.1	183.0	219.3	177.3	**SCSO**	**SCSO**	**SCSO**
**46**	727.2	430.2	287.5	176.9	200.1	137.9	**SCSO**	**SCSO**	**SCSO**
**47**	969.8	526.8	670.6	391.5	227.2	147.0	**SCSO**	**SCSO**	**SCSO**
**48**	840.7	461.0	301.3	187.5	198.9	127.6	**SCSO**	**SCSO**	**SCSO**
**49**	281.3	208.7	311.2	198.4	230.0	170.2	**SCSO**	**SCSO**	**SCSO**
**50**	1376.6	752.8	484.6	307.0	264.4	148.7	**SCSO**	**SCSO**	**SCSO**

^a^Exp. is the abbreviation of experience.

Table 2 presents the statistical results of objective function values and average execution times for three methods in 50 different maps, including 5 statistical indicators: the best value, the worst value, mean value, median value and standard deviation. The optimal results among the three methods are highlighted in bold. All 5 statistical indicators of the SCSO algorithm are superior to other methods, meaning SCSO algorithm has good performance and robustness in ORD. Moreover, the SCSO algorithm also exhibits the shortest average execution time in these experiments.

**Table 2:** Statistical results of the three methods in 50 different maps.

Method	Best	Worst	Mean	Median	Standard deviation	Average execution time (s)
PSO	490.003	4976.057	1377.044	1178.5	726.394	1510.34
DBO	265.852	1062.128	505.3486	472.807	166.4458	1030.23
SCSO	**264.348**	**531.9975**	**382.1134**	**380.8823**	**62.7311**	**521.09**

### Results of outdoor orienteering route design

**Fig 6:**
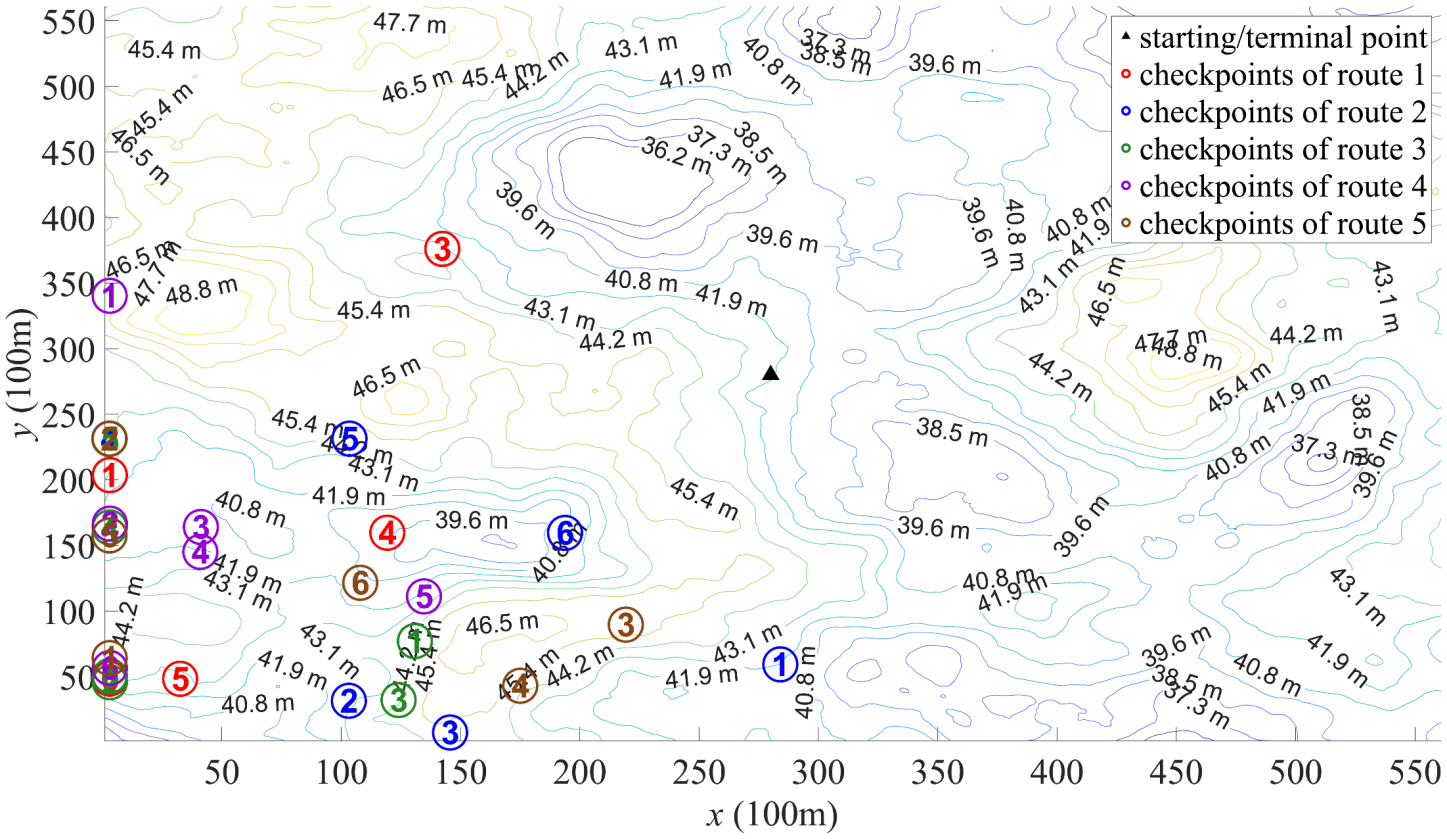
Route designed by PSO algorithm.

**Fig 7:**
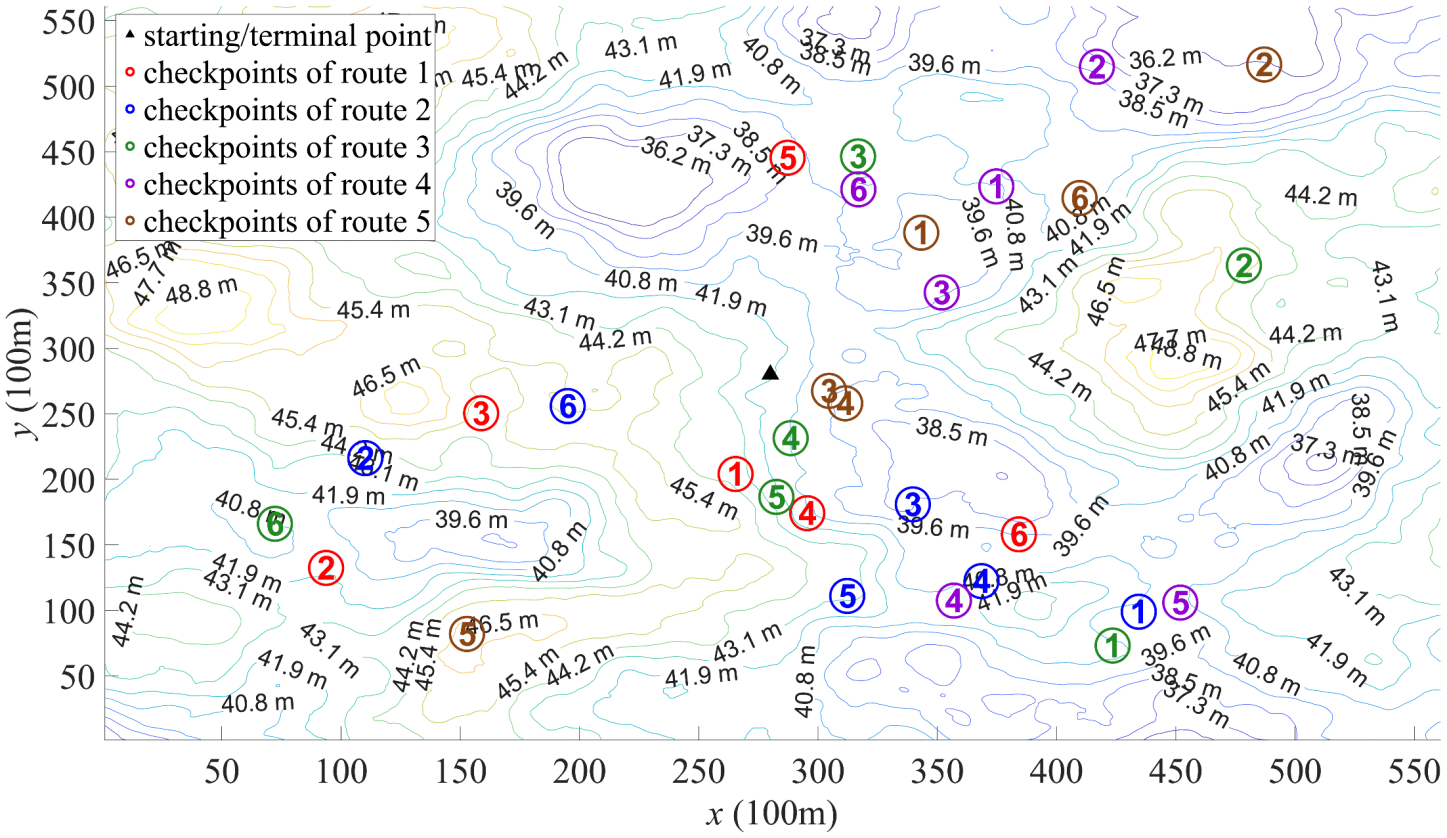
Route designed by DBO algorithm.

**Fig 8:**
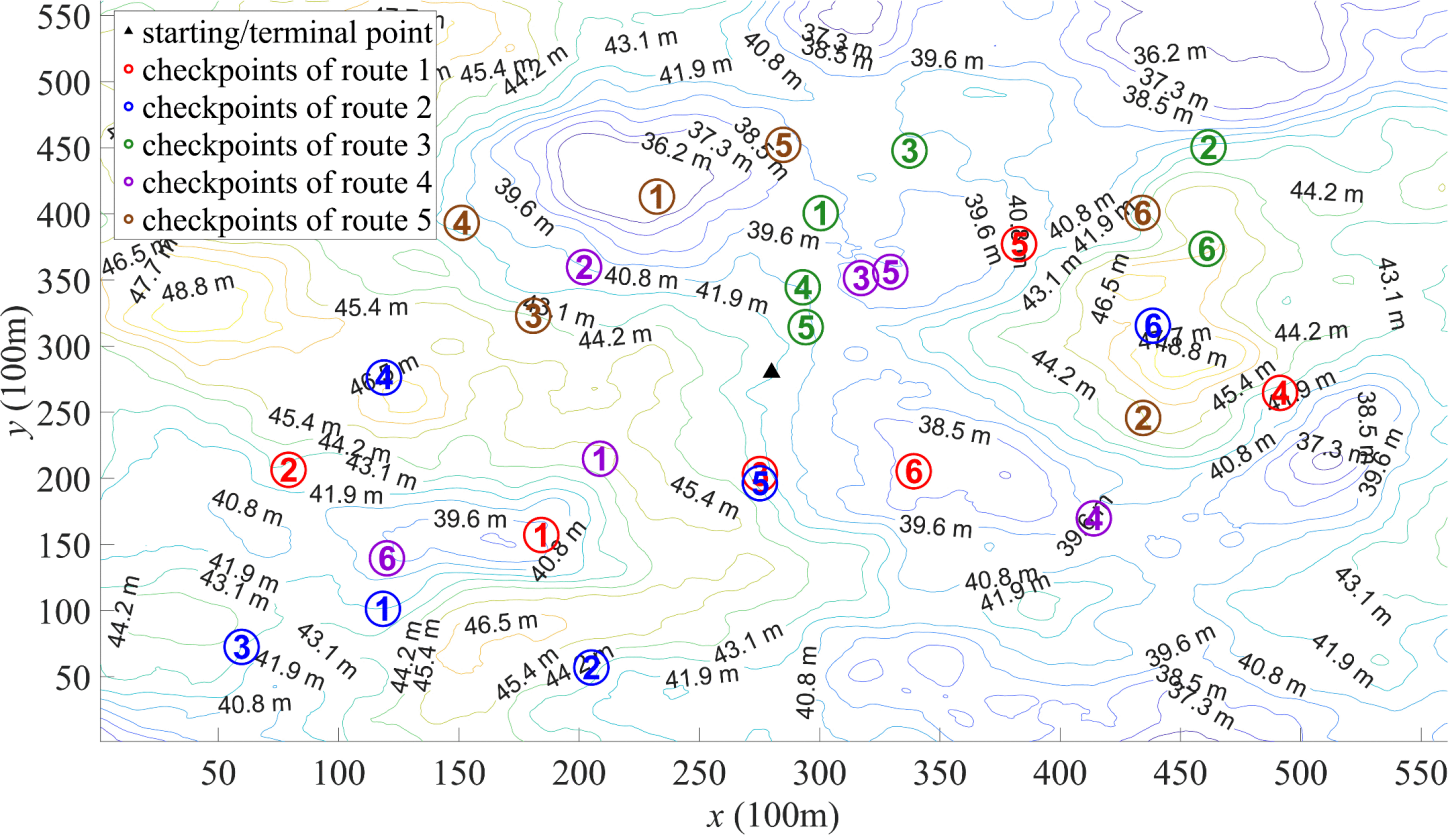
Route designed by SCSO algorithm.

For the map shown in Fig 2, the ORD results are illustrated in Figs 6–8. Fig 6 depicts the route designed by PSO algorithm. The significant variation in total lengths among these routes can lead to unfairness in outdoor orienteering. Moreover, there are pronounced overlaps between some routes, and some checkpoints are overly concentrated, which may impact the orienteering experience of participants. This phenomenon may be attributed to PSO algorithm getting stuck in local optima. Fig 7 depicts the route designed by DSO algorithm. The total lengths of these routes have relatively small differences, and the distances between different checkpoints are also small. However, the distribution of checkpoints in some routes is not sufficiently dispersed (as the route 2 and the route 5), which can affect participants’ orienteering experience. This phenomenon may be due to the insufficient exploratory capabilities of DBO algorithm in ORD. Fig 8 depicts the route designed by SCSO algorithm. The total lengths of these routes and the distances between different checkpoints show minimal differences. The distribution of checkpoints in each route is relatively dispersed, and the overlapping areas between routes are minimal. This indicates that SCSO algorithm possesses strong exploratory capabilities and the ability to escape local optima in ORD, allowing SCSO algorithm to better meet the requirements for justice and participant experience.

## Conclusions

This study establishes five fundamental principles for outdoor orienteering route design to simultaneously ensure competitive fairness and enhance participant experience. To systematically address the ORD challenge, we formulate the problem as a multi-objective optimization framework and implement the sand cat swarm optimization (SCSO) algorithm as the computational engine. Our simulation methodology incorporates 2048 randomly generated convex areas to approximate real-world terrain variability, with inter-checkpoint distances calculated using an artificial potential field (APF) approach rather than Euclidean measurements. Extensive simulations confirm the proposed method’s operational efficiency, practical feasibility, and algorithmic robustness. Comparative evaluations against particle swarm optimization (PSO) and dung beetle optimizer (DBO) algorithms under varied scenarios demonstrate SCSO’s superior performance in ORD applications. The main contributions of this paper are as follows: 1) Development of a principled ORD framework providing actionable design guidelines for orienteering planners. 2) Formalization of ORD as a mathematically tractable optimization problem with effective computational solutions. 3) Implementation of an intelligent ORD system that significantly reduces manual design workload while maintaining competitive integrity.

While the proposed framework demonstrates promising results, several limitations warrant attention. The optimization process excludes expert planner input, potentially overlooking tacit knowledge accumulated through years of practical route design experience. Future research may focus on developing human-AI collaborative design paradigms, particularly through interactive evolutionary algorithms that systematically incorporate real-time feedback from expert planners during the optimization cycle.

## References

[1] Waddington EE, Allison DJ, Calabrese EM, Pekos C, Lee A, Walsh JJ, et al. Orienteering combines vigorous-intensity exercise with navigation to improve human cognition and increase brain-derived neurotrophic factor. PLoS One. 2024;19(5):e0303785. doi: 10.1371/journal.pone.0303785 38776348 PMC11111042

[2] Batista MM, Paludo AC, Gula JN, Pauli PH, Tartaruga MP. Physiological and cognitive demands of orienteering: a systematic review. Sport Sci Health. 2020;16(4):591–600. doi: 10.1007/s11332-020-00650-6

[3] Waddington EE, Heisz JJ. Orienteering experts report more proficient spatial processing and memory across adulthood. PLoS One. 2023;18(1):e0280435. doi: 10.1371/journal.pone.0280435 36662692 PMC9858405

[4] Mukhina KD, Visheratin AA, Nasonov D. Orienteering problem with functional profits for multi-source dynamic path construction. PLoS One. 2019;14(4):e0213777. doi: 10.1371/journal.pone.0213777 30939132 PMC6445411

[5] Sanderud JR, Gurholt KP, Moe VF. Didactic sensitivity to children and place: a contribution to outdoor education cultures. Sport Educ Soc. 2021;27(9):1086–99. doi: 10.1080/13573322.2021.1966409

[6] Elzein A, Di Caro GA. A clustering metaheuristic for large orienteering problems. PLoS ONE. 2022;17(7):e0271751. doi: 10.1371/journal.pone.0271751 PMC9307188 35867693

[7] Muller J, McEwan K, Gorczynski P, Weston N. Defining contemporary outdoor physical activity: a critical interpretive synthesis. J Outdoor Recreat Tour. 2024;47:100799. doi: 10.1016/j.jort.2024.100799

[8] Xu Y, Qian J. Examining the risk-safety paradox in outdoor education from a Taoist perspective: a case study of a Chinese outdoor education experience. Sport Educ Soc. 2023;30(2):137–53. doi: 10.1080/13573322.2023.2295874

[9] Tyne WP, Fletcher D, Paine NJ, Stevinson C. Employees’ experiences of outdoor adventure training on psychological capital and wellbeing: a mixed methods case study. J Outdoor Recreat Tour. 2024;46:100761. doi: 10.1016/j.jort.2024.100761

[10] Mackenzie SH, Son JS, Eitel K. Using outdoor adventure to enhance intrinsic motivation and engagement in science and physical activity: An exploratory study. J Outdoor Recreat Tour. 2018;21:76–86. doi: 10.1016/j.jort.2018.01.008

[11] Schlemmer P, Scholten T, Niedermeier M, Kopp M, Schnitzer M. Do outdoor adventure park activities increase visitors’ well-being? J Outdoor Recreat Tour. 2021;35:100391. doi: 10.1016/j.jort.2021.100391

[12] Pomfret G, Sand M, May C. Conceptualising the power of outdoor adventure activities for subjective well-being: a systematic literature review. J Outdoor Recreat Tourism. 2023;42:100641. doi: 10.1016/j.jort.2023.100641

[13] Freeman NK, Keskin BB, Çapar İ. Attractive orienteering problem with proximity and timing interactions. Eur J Operat Res. 2018;266(1):354–70. doi: 10.1016/j.ejor.2017.09.025

[14] Lam HKN, Sproule J, Turner AP, Murgatroyd P, Gristwood G, Richards H, et al. International orienteering experts’ consensus on the definition, development, cause, impact and methods to reduce mental fatigue in orienteering: a Delphi study. J Sports Sci. 2022;40(23):2595–607. doi: 10.1080/02640414.2023.2177027 36765435

[15] Liang S, Jiao T, Du W, Qu S. An improved ant colony optimization algorithm based on context for tourism route planning. PLoS One. 2021;16(9):e0257317. doi: 10.1371/journal.pone.0257317 34529729 PMC8445481

[16] Li W, Wang G-G, Gandomi AH. A survey of learning-based intelligent optimization algorithms. Arch Computat Methods Eng. 2021;28(5):3781–99. doi: 10.1007/s11831-021-09562-1

[17] Cui Y, Geng Z, Zhu Q, et al. Multi-objective optimization methods and application in energy saving. Energy. 2017;125:681–704. doi: 10.1007/s11831-021-09562-1

[18] Strauss A. A method for complete plant taxon and site inventories in large forest areas with the help of orienteering maps, as exemplified by target forests in Switzerland. PLoS One. 2019;14(12):e0225927. doi: 10.1371/journal.pone.0225927 31821345 PMC6903739

[19] Zhang Y, Gong D, Zhang J. Robot path planning in uncertain environment using multi-objective particle swarm optimization. Neurocomputing. 2013;103:172–85. doi: 10.1016/j.neucom.2012.09.019

[20] Nazarahari M, Khanmirza E, Doostie S. Multi-objective multi-robot path planning in continuous environment using an enhanced genetic algorithm. Exp Syst Appl. 2019;115:106–20. doi: 10.1016/j.eswa.2018.08.008

[21] Patle BK, Babu L G, Pandey A, Parhi DRK, Jagadeesh A. A review: On path planning strategies for navigation of mobile robot. Defence Technol. 2019;15(4):582–606. doi: 10.1016/j.dt.2019.04.011

[22] Seyyedabbasi A, Kiani F. Sand cat swarm optimization: a nature-inspired algorithm to solve global optimization problems. Eng Comput. 2022;39(4):2627–51. doi: 10.1007/s00366-022-01604-x

[23] Yao L, Yang J, Yuan P, Li G, Lu Y, Zhang T. Multi-strategy improved sand cat swarm optimization: global optimization and feature selection. Biomimetics (Basel). 2023;8(6):492. doi: 10.3390/biomimetics8060492 37887623 PMC10604673

[24] Kiani F, Anka FA, Erenel F. PSCSO: enhanced sand cat swarm optimization inspired by the political system to solve complex problems. Adv Eng Softw. 2023;178:103423. doi: 10.1016/j.advengsoft.2023.103423

[25] Szwarc K, Boryczka U. A novel approach to the orienteering problem based on the harmony search algorithm. PLoS One. 2022;17(2):e0264584. doi: 10.1371/journal.pone.0264584 35226706 PMC8884584

[26] Xue J, Shen B. Dung beetle optimizer: a new meta-heuristic algorithm for global optimization. J Supercomput. 2022;79(7):7305–36. doi: 10.1007/s11227-022-04959-6

